# The Impact of Cirrhosis on Outcomes of Patients Admitted With Diabetic Ketoacidosis: A Nationwide Study

**DOI:** 10.7759/cureus.25870

**Published:** 2022-06-12

**Authors:** Mahmoud M Mansour, Adham E Obeidat, Mohammad Darweesh, Ratib Mahfouz, Scott Kuwada, Nikolaos T Pyrsopoulos

**Affiliations:** 1 Internal Medicine, University of Missouri School of Medicine, Columbia, USA; 2 Internal Medicine, University of Hawaii, Honolulu, USA; 3 Internal Medicine, East Tennessee State University, Johnson City, USA; 4 Internal Medicine, Kent Hospital, Brown University, Warwick, USA; 5 Gastroenterology and Hepatology, University of Hawaii, Honolulu, USA; 6 Gastroenterology and Hepatology, Rutgers University New Jersey Medical School, New Jersey, USA

**Keywords:** diabetic ketoacidosis, compensated cirrhosis, decompensated cirrhosis, cirrhosis dka, dka, nationwide inpatient sample (nis), nationwide sample, liver cirrhosis, cirrhosis

## Abstract

Introduction

Diabetic ketoacidosis (DKA) is the most common acute hyperglycemic emergency in people with diabetes mellitus (DM). Cirrhosis is a consequence of chronic inflammation that is followed by hepatic fibrosis. It has been noted that cirrhosis is associated with an increased risk of developing type II DM due to altered glucose homeostasis. The prognostic value of DM in cirrhotic patients has been studied before and was found to be associated with lower survival. However, the risk of mortality and adverse events in cirrhotic patients admitted with DKA needs further evaluation. The aim of this study is to compare outcomes in patients with cirrhosis admitted to the hospital with DKA compared to non-cirrhotic patients.

Methods

The data for this study were extracted from the National Inpatient Sample (NIS) 2016-2019. The NIS was queried for all patients who had a discharge diagnosis of DKA. Patients with cirrhosis were identified and subclassified into compensated and decompensated cirrhosis using the International Classification of Diseases 10th revision, Clinical Modification (ICD-10-CM) codes. Patients without cirrhosis were the control group. ICD-10-CM codes that have been validated for cirrhosis were utilized. The primary outcome was in-hospital mortality. Secondary outcomes were hospital charges, length of stay (LOS), and in-hospital complications, including shock, mechanical ventilation, and acute kidney injury (AKI) requiring dialysis.

Results

We included 1,098,875 hospitalizations with a discharge diagnosis of DKA. Overall, 9,190 patients had compensated cirrhosis and 4,355 had decompensated cirrhosis. Cirrhotic patients had overall worse outcomes compared to non-cirrhotics. Decompensated cirrhotics had the highest mortality (11.26%; 95% confidence interval [CI]: 9.36% to 13.49%) compared to compensated cirrhotics (3.54%; 95% CI: 2.79% to 4.48%) and non-cirrhotics (2.15%; 95% CI: 1.89% to 2.43%). Similarly, decompensated cirrhotics also had the highest LOS, total charges, and in-hospital complications among the groups. On multivariate analysis, decompensated cirrhosis, rather than compensated cirrhosis, was an independent predictor of higher mortality (adjusted odds ratio [AOR]: 2.30; 95% CI: 1.81 to 2.92), LOS (regression coefficient: +1.82 days; 95% CI: +1.19 to +2.44 days), hospital charges (regression coefficient: +$28,497; 95% CI: +$18,107 to +$38,887), shock (AOR: 2.31; 95% CI: 1.68 to 3.18), mechanical ventilation (AOR: 1.91; 95% CI: 1.58 to 2.29), and AKI requiring dialysis (AOR: 2.31; 95% CI: 1.68 to 3.18).

Conclusion

This study showed that patients with decompensated liver cirrhosis who were admitted with DKA had the worst in-hospital outcomes. Additionally, only decompensated cirrhosis was an independent predictor of worse outcomes. Decompensated cirrhotics who develop DKA should be approached with more caution with a probable lower threshold for intensive care unit admission for a higher level management.

## Introduction

Diabetic ketoacidosis (DKA) is the most common acute hyperglycemic emergency in people with diabetes mellitus (DM) [1]. This potentially life-threatening complication can occur in both type 1 and type 2 DM, and it can be associated with significant morbidity and increased length of hospital stay [2,3]. Mortality rates in DKA patients have fallen significantly in the past 20 years to <1% [3,4].

Liver cirrhosis is a consequence of chronic liver inflammation followed by diffuse hepatic fibrosis, which can eventually lead to liver failure [5]. Cirrhosis is the leading cause of liver-related death globally. Deaths due to cirrhosis constituted 2.4% of total deaths globally in 2017 compared with 1.9% in 1990 [6]. Age-standardized deaths due to cirrhosis are highest in Egypt due to the high prevalence of hepatitis C and hepatitis B and lowest in Singapore [6]. The natural history of cirrhosis starts with an asymptomatic phase known as compensated cirrhosis, followed by the development of complications from portal hypertension and/or liver dysfunction, termed as decompensated cirrhosis [1]. Most deaths in patients with decompensated cirrhosis result from hepatic and extrahepatic organ failure. Deaths during the compensated stage are largely due to cardiovascular disease, malignancy, and renal disease [5].

Cirrhosis is associated with an increased risk of developing type II DM due to altered glucose homeostasis [7]. The prognostic value of DM in cirrhotic patients has been studied before and was found to be associated with lower survival [7-11]. However, to the best of our knowledge, no studies have been conducted before to evaluate the risk of mortality and adverse events in cirrhotic patients admitted with DKA compared to non-cirrhotic patients. This national inpatient-based study will compare potential outcomes in patients with liver cirrhosis admitted to the hospital with DKA with outcomes in non-cirrhotic DKA patients.

## Materials and methods

Data source

The data for this study were extracted from the National Inpatient Sample (NIS) 2016-2019. NIS is the largest publicly available all-payer inpatient healthcare database [12]. The NIS is drawn from all states participating in the Healthcare Cost and Utilization Project (HCUP), covering more than 97% of the United States population. The database contains data on more than seven million hospital stays with a weighted estimate of 35 million hospital stays each year. It includes information on patients’ demographics, hospital characteristics, hospital outcomes, and up to 40 diagnostic and 25 procedure codes based on the International Classification of Diseases 10th revision, Clinical Modification (ICD-10-CM), and Procedure Coding System (ICD-10-PCS).

The University of Missouri School of Medicine Institutional Review Board has deemed research using the NIS and similar deidentified datasets exempt from institutional approval.

Study design and inclusion criteria

The NIS was queried for all patients who had a discharge diagnosis of DKA between 2016 and 2019. Patients younger than 18 years, elective admissions, and patients with missing demographic variables such as age, gender, and age were excluded from the study. Patients with cirrhosis were identified and subclassified into compensated and decompensated cirrhosis. Compensated cirrhotics were identified using ICD-10 codes for cirrhosis without the presence of decompensation variables: portal hypertension, hepatic encephalopathy, ascites, variceal bleeding, hepatocellular carcinoma, or hepatorenal syndrome. Decompensated cirrhosis was defined using both cirrhosis diagnosis and one or more decompensation variables. Combination codes for defining compensated and decompensated cirrhosis have been validated and shown to have a good positive and negative predictive value [13]. Patients without cirrhosis were considered the control group. Table 1 contains the list of ICD-10-CM and ICD-10-PCS codes used in the study.

**Table 1:** ICD-10 codes used in the study ICD-10, International Classification of Diseases, 10th revision

Variables	ICD-10 codes
Cirrhosis variables	K70.30, K74.60, K74.69
Decompensation variables	K7040, K70.41, K72.00, K72.10, K72.01, K72.11, K72.90, K72.91, K70.31, K76.7, K76.81, I85.01, I85.11, K652, R18.8, C2.20
Compensated cirrhosis	Any of the cirrhosis variables without the decompensation variables
Decompensated cirrhosis	Any of the cirrhosis variables + one of the decompensation cirrhosis variables
Mechanical ventilation	5A1935Z, 5A1945Z, 5A1955Z
Shock requiring vasopressor use	3E030XZ, 3E033XZ, 3E040XZ, 3E043XZ, 3E050XZ, 3E053XZ, 3E060XZ,3E063XZ
Acute kidney injury requiring dialysis	One of the following: N17.0, N17.1, N17.2, N17.8, N17.9 + one of the following: 5A1D70Z, 5A1D80Z, 5A1D90Z

Patients’ demographics included in the NIS database were age, sex, race, median household income, and insurance status. Hospital characteristics included hospital size, region, and teaching status. The comorbidity burden was assessed using the Charlson comorbidity index. The primary outcome was in-hospital mortality. Secondary outcomes were hospital charges, length of stay (LOS), and the following in-hospital complications: mechanical ventilation, vasopressor use, and acute kidney injury (AKI) requiring dialysis.

Statistical analysis

Data analysis was performed using Stata version 17 (StataCorp, College Station, TX). This software implements weighing of patient-level observations, which facilitates producing nationally representative unbiased results, variance estimates, and p-values. Statistical hypotheses were tested using p < 0.05 as the level of statistical significance. Categorical variables were compared using the chi-square test, and continuous variables were compared using Student’s t-test. Outcomes were evaluated using a multivariate logistic regression model to adjust for confounders. Confounders included in the multivariate regression test were the Charlson comorbidity index along with patients’ demographics and hospital characteristics. Covariates with p ≥ 0.1 on univariate analysis, except for age and sex, were excluded from the multivariate analysis.

## Results

Patient and hospital characteristics

We included 1,098,875 weighted discharges of DKA between January 1, 2016, and December 30, 2019. Of those, 9,190 patients had compensated cirrhosis and 4,355 had decompensated cirrhosis. Table 2 details the characteristics of the control group (non-cirrhotic patients with DKA), compensated cirrhotics with DKA, and decompensated cirrhotics with DKA. The cirrhotic groups were older (mean age of 54.82 and 56.31 years in the compensated and decompensated groups, respectively, vs. 45.08 years in the non-cirrhotics, p < 0.001). Cirrhotic patients were predominantly males (60.80% and 62.80% in the compensated and decompensated groups, respectively, vs. 50.31% in the non-cirrhotic group, p < 0.001). Patients with cirrhosis were less likely to be black (15.34% and 12.63% in the compensated and decompensated groups, respectively, vs. 24.95% in the non-cirrhotic group, p < 0.001) and more likely to be Hispanic (17.95% and 22.50% in the compensated and decompensated groups, respectively, vs. 12.61% in the non-cirrhotic group, p < 0.001). In addition, cirrhotic patients were more likely to have Medicare insurance and were more likely to be admitted to teaching hospitals. There was no difference in income between the groups.

**Table 2:** Patient characteristics in patients admitted with diabetic ketoacidosis: without cirrhosis (control group), with compensated cirrhosis, and with decompensated cirrhosis

Characteristics	No cirrhosis	Compensated cirrhosis	p-Value	Decompensated cirrhosis	p-Value
Female (%)	49.69%	39.20%	<0.001	37.20%	<0.001
Mean age (years)	45.08	54.82		56.31	
Race (%)			<0.001		<0.001
White	57.36%	59.74%		57.75%	
Black	24.95%	15.34%		12.63%	
Hispanic	12.61%	17.95%		22.50%	
Asian or Pacific Islander	1.51%	1.36%		1.72%	
Native American	1.02%	2.72%		2.99%	
Other	2.55%	2.88%		2.41%	
Charlson comorbidity index			<0.001		<0.001
0	0.14%	0.00%		0.00%	
1	43.58%	0.27%		0.11%	
2	24.56%	26.01%		8.15%	
3	9.55%	20.51%		6.08%	
≥4	22.17%	53.21%		85.65%	
Income			0.113		0.121
1-47,999	38.27%	37.93%		35.45%	
48,000-60,999	27.15%	25.04%		26.88%	
61,000-81,999	21.21%	23.06%		21.71%	
82,000+	13.37%	13.96%		15.96%	
Insurance			<0.001		<0.001
Medicare	29.38%	42.04%		40.91%	
Medicaid	32.48%	33.82%		33.09%	
Private insurance	26.65%	17.39%		18.29%	
Self-pay	11.50%	6.75%		7.70%	
Region of hospital			<0.001		<0.001
Northeast	15.21%	14.31%		14.70%	
Midwest	20.80%	18.39%		17.68%	
South	43.75%	41.62%		38.58%	
West	20.23%	25.68%		29.05%	
Hospital size			0.070		0.0120
Small	21.70%	21.16%		17.91%	
Medium	30.22%	28.07%		29.62%	
Large	48.08%	50.76%		52.47%	
Location/ teaching status			0.005		<0.001
Rural	10.30%	8.38%		5.51%	
Urban, nonteaching	23.15%	21.65%		19.86%	
Urban, teaching	66.55%	69.97%		74.63%	

In-hospital mortality

**Figure 1:**
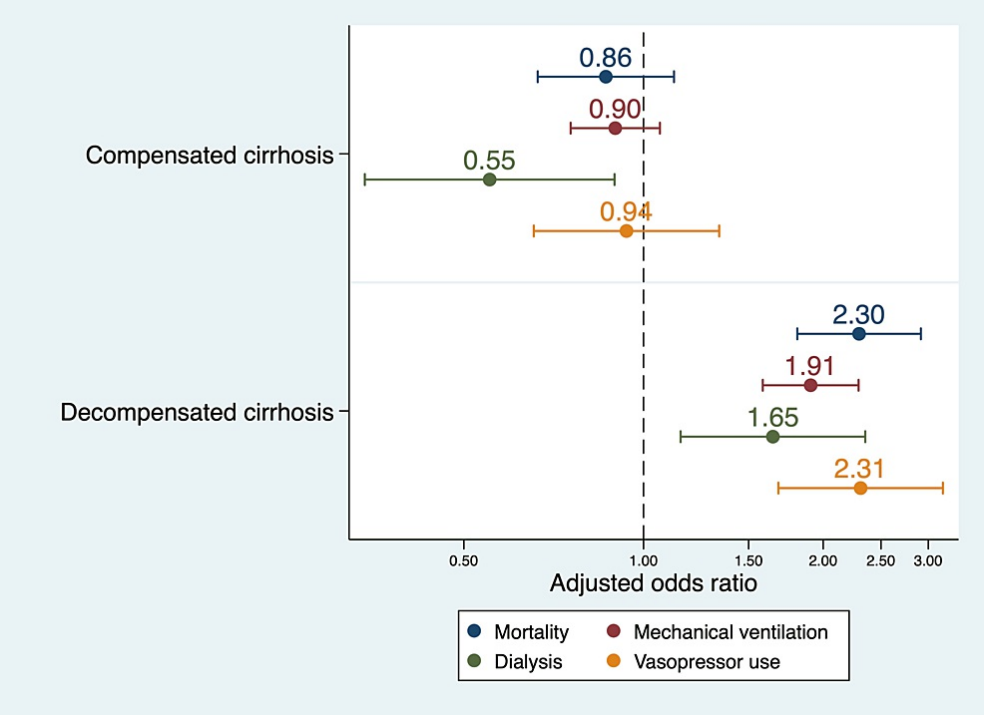
Forest plot showing adjusted odds ratio for categorical outcomes in compensated and decompensated cirrhosis

In-hospital mortality in non-cirrhotics with DKA was 2.15% (95% confidence interval [CI]: 1.89% to 2.43%). The total mortality was 3.54% (95% CI: 2.79% to 4.48%) in compensated cirrhotics and 11.26% (95% CI: 9.36% to 13.49%) in decompensated cirrhotics. On multivariate analysis, there was no significant difference between in-hospital mortality in the compensated cirrhotic group (adjusted odds ratio [AOR]: 0.86; 95% CI: 0.66 to 1.12). However, those with decompensated cirrhosis had significantly higher mortality (AOR: 2.30; 95% CI: 1.81 to 2.92). Table 3 summarizes the outcomes in the study groups. Figure 1 shows a forest plot of AOR of the studied categorical outcomes.

**Table 3:** In-hospital outcomes of patients with diabetic ketoacidosis: no cirrhosis (control group) vs. compensated cirrhosis vs. decompensated cirrhosis AOR, adjusted odds ratio

Outcomes	Study population with diabetic ketoacidosis, n = 1,098,875		
	No cirrhosis (control group), n = 1,085,330	Compensated cirrhosis, n = 9,190	Decompensated cirrhosis, n = 4,355
In-hospital mortality	2.15%	3.54% (p = 0.001)	11.26% (p < 0.001)
AOR	Ref	0.86 (p = 0.279)	2.30 (p < 0.001)
Mean length of stay (days)	4.65	6.00 (p < 0.001)	9.01 (p < 0.001)
Adjusted coefficient	Ref	-0.13 (p = 0.418)	+1.82 (p < 0.001)
Mean hospital charges	$52,650	$69,611 (p < 0.001)	$120,118 (p < 0.001)
Adjusted coefficient	Ref	-$6,968 (p = 0.004)	+$28,497 (p = 0.004)
Mechanical ventilation	6.15%	9.30% (p < 0.001)	21.47% (p < 0.001)
AOR	Ref	0.90 (p = 0.214)	1.91 (p < 0.001)
Shock requiring vasopressors	1.11%	1.74% (p < 0.014)	5.40% (p < 0.001)
AOR	Ref	0.93 (p = 0.718)	2.31 (p < 0.001)
Acute kidney injury requiring dialysis	0.81%	0.92% (p = 0.632)	4.25% (p < 0.001)
AOR	Ref	0.55 (p = 0.016)	1.65 (p = 0.006)

Mean hospitalization charges

The mean hospitalization charges for non-cirrhotic patients admitted with DKA was $52,650 (95% CI: $51,880 to $53,420). Mean hospitalization charges for compensated and decompensated cirrhotics were $69,611 (95% CI: $65,013 to $74,210) and $120,118 (95% CI: $108,753 to $131,483), respectively. When adjusted with multivariate logistic regression, the difference in mean hospitalization charges was significantly higher in the decompensated cirrhotics group (+$28,497; 95% CI: +$18,107 to +$38,887) and slightly lower in the compensated cirrhosis group (-$6,968; 95% CI: -&5575 to -$8362).

Length of stay

The mean LOS for non-cirrhotic patients admitted with DKA was 4.65 days (95% CI: 4.62 to 4.69 days). Mean LOS for compensated and decompensated cirrhotics was 6.00 days (95% CI: 5.71 to 6.29 days) and 9.01 days (95% CI: 8.36 to 9.66 days), respectively. On multivariate logistic regression, patients with decompensated cirrhosis had longer mean LOS (+1.82 days; 95% CI: +1.19 to +2.44 days), while patients with compensated cirrhosis did not have a statistical difference in LOS (-0.13 days; 95% CI: -0.43 to +0.18 days).

Mechanical ventilation

Of the patients with DKA, 6.15% (CI, 6.03% to 6.27%) were mechanically ventilated in the non-cirrhotics group. Decompensated cirrhotics had a much higher rate of intubation (21.47 %; CI 18.87% to 24.32%) as opposed to compensated cirrhotics who had a slightly higher rate of intubation (9.30%; CI, 8.05% to 10.72%). After adjusting for confounders, compensated cirrhotics did not have a significant increase in mortality while decompensated cirrhotics did (AOR, 0.90; CI 0.75 to 1.07 and 1.91; CI, 1.58 to 2.29, respectively).

Shock requiring vasopressors

Of the patients with DKA, 1.11 % (95% CI: 1.04% - 1.18%) developed shock requiring vasopressors in the non-cirrhotics group. Compensated cirrhotics had a slightly higher rate of vasopressor use (1.74%; 95% CI: 1.22% to 2.47%), while decompensated cirrhotics had a much higher rate of vasopressor use (5.40%; 95% CI: 4.09% to 7.09%). After adjusting for confounders, only decompensated cirrhotics had a significant increase in vasopressor use (AOR: 2.31; 95% CI: 1.68 to 3.18), while compensated cirrhotics did not (AOR: 0.93; 95% CI: 0.65 to 1.34).

Acute kidney injury requiring dialysis

Of the patients with DKA, 0.81% (95% CI: 0.77% to 0.86%) developed AKI requiring dialysis in the non-cirrhotics group. Compensated cirrhotics did not have a significant increase in AKI requiring dialysis (0.92%; 95% CI: 0.58% to 1.48%), while those with decompensated cirrhosis did (4.25%; 95% CI: 3.1% to 5.80%). When adjusted for confounders, decompensated cirrhotics had higher odds of developing AKI with dialysis (1.65; 95% CI: 1.15 to 2.35). Interestingly, compensated cirrhotics had lower adjusted odds for developing AKI requiring dialysis (0.55; 95% CI: 0.34 to 0.89).

## Discussion

This study showed that patients with liver cirrhosis admitted with DKA had overall worse outcomes than non-cirrhotics, with decompensated cirrhotics having the worst outcomes of all groups. Decompensated liver cirrhosis in patients admitted with DKA was associated with significantly increased mortality, length of hospital stay, and higher hospital charges. Moreover, decompensated cirrhotics had increased in-hospital complications such as an increased risk of respiratory failure requiring mechanical ventilation, AKI requiring dialysis, and shock requiring vasopressors. On the other hand, although patients with compensated liver cirrhosis had worse mortality, increased rates of respiratory failure requiring mechanical ventilation, and shock requiring vasopressors, this association was not significant on multivariate analysis. These findings indicate that decompensated cirrhosis, rather than compensated cirrhosis, is an independent predictor of worse outcomes in patients admitted with DKA. Interestingly, after controlling for other factors, patients with compensated liver cirrhosis had lower odds of developing an AKI requiring dialysis compared to non-cirrhotics.

There is no well-established explanation for the worse mortality outcomes in patients with decompensated liver cirrhosis admitted with DKA compared to non-cirrhotics. However, this can be potentially attributed to the poor liver function in decompensated cirrhotic patients, which, in turn, will affect glucose metabolism and homeostasis. Several structural changes in liver cirrhosis can decrease the extraction of insulin by the liver, leading to increased systemic insulin levels by a reduction in liver cell mass and/or the formation of portosystemic venous collaterals [14,15]. This resultant hyperinsulinemia can lead to resistance to insulin through insulin receptor downregulation [16]. Other described mechanisms of liver cirrhosis effect on glucose homeostasis include increased advanced glycations products, increased formation of hypoxia-inducible factors, and reduced pancreatic beta-cell function, which will lead to decreased insulin secretion [17-19]. All of these mechanisms will lead to increased insulin resistance in cirrhotic patients and thus may contribute to worse outcomes of patients who develop DKA. Such changes are expected to be more pronounced in patients with decompensated liver cirrhosis compared to patients with compensated cirrhosis due to advanced stages of liver fibrosis and functional impairment.

Our study has also shown that patients with decompensated liver cirrhosis admitted with DKA are at a very high risk of developing respiratory failure requiring mechanical ventilation. Patients with liver cirrhosis are at an increased risk of respiratory complications such as hepatopulmonary syndrome (HPS), portopulmonary hypertension, and hepatic hydrothorax, which, in turn, can increase the risk of respiratory failure and mechanical ventilation [20]. A prospective study conducted by Schenk et al. on 111 patients with liver cirrhosis, including 27 patients with HPS, showed that patients with HPS had a higher mean Child-Pugh score and MELD (Model for End-Stage Liver Disease) score [21]. Moreover, they found that arterial deoxygenation was more severe in patients with a higher Child-Pugh class [21]. This can explain our finding that patients admitted with DKA who also have decompensated cirrhosis have a higher chance of respiratory failure and mechanical ventilation compared to non-cirrhotics.

Our study has some limitations. Due to the nature of the NIS database, our observations reflect admissions and not individual patients. Therefore, the unit of analysis is the admission. Given the inability to account for multiple admissions for a given patient in the NIS, our conclusions may be confounded by the risk of repeat hospitalization. Thus, our reported rates may be viewed as overestimates of a per-patient admission rate. Mortality rates, however, are unlikely to be affected. Under- or over-coding can lead to misclassification, although the large number of patients in the database strongly mitigates against substantial misclassification bias. NIS undergoes data quality assessment annually to ensure the internal validity of the data. Moreover, patients in the decompensated group were not classified based on the etiology of decompensation, as this can potentially affect the outcome. Additionally, observational studies may not be able to fully adjust for unmeasured confounding factors that might affect our estimates for the reported associations between in-hospital mortality and included covariates. Therefore, conclusions based on these observational data should be viewed as associational and not causal in nature. Finally, these observations pertain to the DKA population in the United States and may not be generalizable to other cirrhosis populations in other countries.

## Conclusions

This study showed that patients admitted with DKA who also have decompensated cirrhosis had the worst outcomes compared to those with compensated cirrhosis and without cirrhosis. Furthermore, decompensated cirrhosis was an independent predictor of worse outcomes in DKA patients. DKA should be approached with more caution in decompensated cirrhotics with probable lower threshold for intensive care unit admission for a higher level management.

## References

[1] Kitabchi AE, Umpierrez GE, Miles JM, Fisher JN (2009). Hyperglycemic crises in adult patients with diabetes. Diabetes Care.

[2] Ooi E, Nash K, Rengarajan L (2021). Clinical and biochemical profile of 786 sequential episodes of diabetic ketoacidosis in adults with type 1 and type 2 diabetes mellitus. BMJ Open Diabetes Res Care.

[3] Evans K (2019). Diabetic ketoacidosis: update on management. Clin Med (Lond).

[4] Kichloo A, El-Amir Z, Wani F, Shaka H (2022). Hospitalizations for ketoacidosis in type 1 diabetes mellitus, 2008 to 2018. Proc (Bayl Univ Med Cent).

[5] Schuppan D, Afdhal NH (2008). Liver cirrhosis. Lancet.

[6] GBD 2017 Cirrhosis Collaborators (2020). The global, regional, and national burden of cirrhosis by cause in 195 countries and territories, 1990-2017: a systematic analysis for the Global Burden of Disease Study 2017. Lancet Gastroenterol Hepatol.

[7] Nishida T (2017). Diagnosis and clinical implications of diabetes in liver cirrhosis: a focus on the oral glucose tolerance test. J Endocr Soc.

[8] Garcia-Compean D, Jaquez-Quintana JO, Gonzalez-Gonzalez JA, Maldonado-Garza H (2009). Liver cirrhosis and diabetes: risk factors, pathophysiology, clinical implications and management. World J Gastroenterol.

[9] Kumar R (2018). Hepatogenous diabetes: an underestimated problem of liver cirrhosis. Indian J Endocrinol Metab.

[10] El-Serag HB, Everhart JE (2002). Diabetes increases the risk of acute hepatic failure. Gastroenterology.

[11] El-Serag HB, Tran T, Everhart JE (2004). Diabetes increases the risk of chronic liver disease and hepatocellular carcinoma. Gastroenterology.

[12] (2022). Overview of the National (Nationwide) Inpatient Sample (NIS). http://www.hcup-us.ahrq.gov/nisoverview.jsp.

[13] Lapointe-Shaw L, Georgie F, Carlone D (2018). Identifying cirrhosis, decompensated cirrhosis and hepatocellular carcinoma in health administrative data: A validation study. PLoS One.

[14] Elkrief L, Rautou PE, Sarin S, Valla D, Paradis V, Moreau R (2016). Diabetes mellitus in patients with cirrhosis: clinical implications and management. Liver Int.

[15] Singh MK, Das BK, Choudhary S, Gupta D, Patil UK (2018). Diabetes and hepatocellular carcinoma: a pathophysiological link and pharmacological management. Biomed Pharmacother.

[16] Hall C, Yu H, Choi E (2020). Insulin receptor endocytosis in the pathophysiology of insulin resistance. Exp Mol Med.

[17] Abdel-Razik A, Mousa N, Zakaria S (2020). Advanced glycation end products as a predictor of diabetes mellitus in chronic hepatitis C-related cirrhosis. Front Med (Lausanne).

[18] Gonzalez FJ, Xie C, Jiang C (2018). The role of hypoxia-inducible factors in metabolic diseases. Nat Rev Endocrinol.

[19] Grancini V, Trombetta M, Lunati ME (2015). Contribution of β-cell dysfunction and insulin resistance to cirrhosis-associated diabetes: role of severity of liver disease. J Hepatol.

[20] Benz F, Mohr R, Tacke F, Roderburg C (2020). Pulmonary complications in patients with liver cirrhosis. J Transl Int Med.

[21] Schenk P, Schöniger-Hekele M, Fuhrmann V, Madl C, Silberhumer G, Müller C (2003). Prognostic significance of the hepatopulmonary syndrome in patients with cirrhosis. Gastroenterology.

